# Grand Challenges in Nutrition and Environmental Sustainability

**DOI:** 10.3389/fnut.2014.00003

**Published:** 2014-03-20

**Authors:** Barbara Burlingame

**Affiliations:** ^1^Nutrition Division, Food and Agriculture Organization of the United Nations, Rome, Italy

**Keywords:** nutrition, biodiversity, sustainable diets, environment, food

Nutrition has always straddled sectors, health and agriculture being the obvious two. The environment sector is a logical and important partner sector for nutrition, although not often recognized as such.

Informally and incidentally, nutritionists have included environment sector issues in their research and practice. For example, food composition databases occasionally turn up nutrient data on food biodiversity (1). Food-based dietary guidelines sometime include a recommendation to decrease environmental footprints by eating locally, the Mediterranean Diet Pyramid being one such example (2).

Other activities are more specific. For example, Total Diet Studies, which analyze environmental contaminants in the food supply, are conducted periodically in many countries (3).

Recent formalized manifestations include the cross-cutting initiative on biodiversity for food and nutrition (4), sustainable diets (5), and sustainable consumption and production (6). As new as it all seems, reference and inferences linking nutrition and environmental sustainability can be found going back millennia in the writings and teachings of doctors and philosophers. If we look back nearly a century and a half we find one of the earliest university-level nutrition programs, developed by Ellen Swallow at the Massachusetts Institute of Technology in the late nineteenth century. A nutrition pioneer, Swallow called her subject Human Ecology. Fundamental to this course was the principle that human health and environmental health were linked, with food and nutrition being the key connecting forces (7). Modern iterations of Human Ecology often do not include nutrition, unfortunately.

The era of industrial agriculture emerged in the twentieth century and environmental sustainability was uncoupled from human health. Nutrition became a clinical subject, nutrients became medicine. Meanwhile, food and agriculture became focused on dietary energy production, protein, and little else (8).

In the mid-1980s, there were some efforts to bring environmental sustainability back into the nutrition domain with the work of Gussow and Clancy on dietary guidelines for sustainability (9), but it took another 20+ years, and a host of actual and looming perils for this to take root in mainstream nutrition thinking (10).

The agriculture sector holds much of the blame for environment problems. Industrial agriculture, intensifying production of high-yield starchy staple through monoculture agriculture, leading to significant loss of food biodiversity; excessive use of agricultural chemicals to extract more dietary energy from every hectare while contaminating the very food it produces, along with groundwater and the soil; the greenhouse gas emissions from livestock industries to feed the ever-increasing demand for meat and dairy products. Global gatherings, from the Conference of the Parties of the Convention on Biological Diversity to the Intergovernmental Panel on Climate Change, raise alarms which are too often specific to the environment sector and ignored by agriculture. And global gatherings from the World Food Summit to the World Health Assembly raise alarms about dietary patterns, undernutrition, obesity and micronutrient malnutrition, which are too often specific only to health and fail to embrace the environmental implications. Occasionally the sectors come together to explore their common issues, e.g., the Commission on Genetic Resources for Food and Agriculture addressing key issues in nutrition (11). Here and elsewhere, it is acknowledged that multi-sectoral solutions are required in order to avert collateral damage in one sector caused by policies and programs in another.

With the world’s population predicted to reach 9 billion by 2015, “sustainable production intensification” is one of the catch-cries. But how should it be defined and put into practice? One pragmatic suggestion is to approach it through a nutrition lens and define sustainable production intensification as, “nutrition-driven agriculture within planetary boundaries” (12).

Policies and actions at all levels require more and better intersectoral research to simultaneously address nutrition and environmental sustainability. Mistakes of the past need to be corrected, present day problems need solutions, and the future urgently needs protection. This is truly a frontier in nutrition.
